# Influence of Structural Parameters on Performance of SAW Resonators Based on 128° YX LiNbO_3_ Single Crystal

**DOI:** 10.3390/nano12122109

**Published:** 2022-06-19

**Authors:** Wenping Geng, Caiqin Zhao, Feng Xue, Xiaojun Qiao, Jinlong He, Gang Xue, Yukai Liu, Huifen Wei, Kaixi Bi, Linyu Mei, Xiujian Chou

**Affiliations:** 1 Science and Technology on Electronic Test and Measurement Laboratory, North University of China, Taiyuan 030051, China; s1906211@st.nuc.edu.cn (C.Z.); s1906060@st.nuc.edu.cn (F.X.); xiaojunqiao@nuc.edu.cn (X.Q.); s1906190@st.nuc.edu.cn (J.H.); s1906061@st.nuc.edu.cn (G.X.); sliuyukai@163.com (Y.L.); b1806002@st.nuc.edu.cn (H.W.); bikaixi@nuc.edu.cn (K.B.); chouxiujian@nuc.edu.cn (X.C.); 2 School of Mechanical Engineering, North University of China, Taiyuan 030051, China; mly81@163.com

**Keywords:** surface acoustic wave (SAW), return loss (RL), resonant frequency, resonance device, LiNbO_3_ single crystal

## Abstract

The seeking of resonator with high Q and low insertion loss is attractive for critical sensing scenes based on the surface acoustic wave (SAW). In this work, 128° YX LiNbO_3_-based SAW resonators were utilized to optimize the output performance through IDT structure parameters. Once the pairs of IDTs, the acoustic aperture, the reflecting grid logarithm, and the gap between IDT and reflector are changed, a better resonance frequency of 224.85 MHz and a high Q of 1364.5 were obtained. All the results demonstrate the structure parameters design is helpful for the performance enhancement with regard to SAW resonators, especially for designing and fabricating high-Q devices.

## 1. Introduction

Recently, surface acoustic wave (SAW) resonators have been playing an important role as key devices in a wide group since the Rayleigh wave was first proposed by Lord Rayleigh in 1885 [1]. The resonators based on SAW technology have been reported in various areas including wideband bandpass filters, high sensitivity sensors, and radio frequency (RF) oscillator [2,3,4]. The SAW-based devices show noticeable features of high sensitivity and good stability which are closely related with the Q value and insertion loss [5]. A lot of research has been focused on optimizing materials and structure to enhance the performance of the SAW resonators, which has a certain degree of enlightenment for manufacturing high-Q resonators. As shown in the previous literature, Lu et al. extracted a novel electrode-area-weighted (EAW) method of implementing wavelet transform processor (WTP) with SAW device based on X-112°Y LiTaO_3_ [6]. A. J. Vigil et al. presented approaches for SAW filter design used in pulsed Quadrature Binary Modulation (QBM) systems with split interdigital transducer (IDT) [7]. Ye et al. reported YZ-cut LiNbO_3_ is suitable for the fabrication of SAW temperature sensors for its higher sensitivity and larger Q factor [8]. However, the electromechanical coupling coefficients (K^2^) and phase velocity of X-112°Y LiTaO_3_ and YZ-cut LiNbO_3_ are limited; moreover, the temperature stability of them is relatively poor [9]. Regarding these aspects, 128° YX LiNbO_3_ is a better substrate choice to be utilized for SAW resonator, due to extremely low sound loss and piezoelectric properties. Inspired by the previous work, improving the IDT structure of 128° YX LiNbO_3_-based SAW resonators is meaningful for high Q value and low insertion of SAW resonators.

In this work, 128° YX LiNbO_3_-based SAW resonators were designed and fabricated with different parameters based on equivalent circuit model. Vector network analyzer (VNA) and RF probe station were used to test the signal of the manufactured devices. The comparison and analysis of the influence of different parameters on SAW resonant is performed through experiment results and the SAW resonator with Q 1364.5 is obtained that could be used for high performance SAW devices preparation.

## 2. Experimental

### 2.1. Design and Materials of SAW Resonator

**Figure 1:**
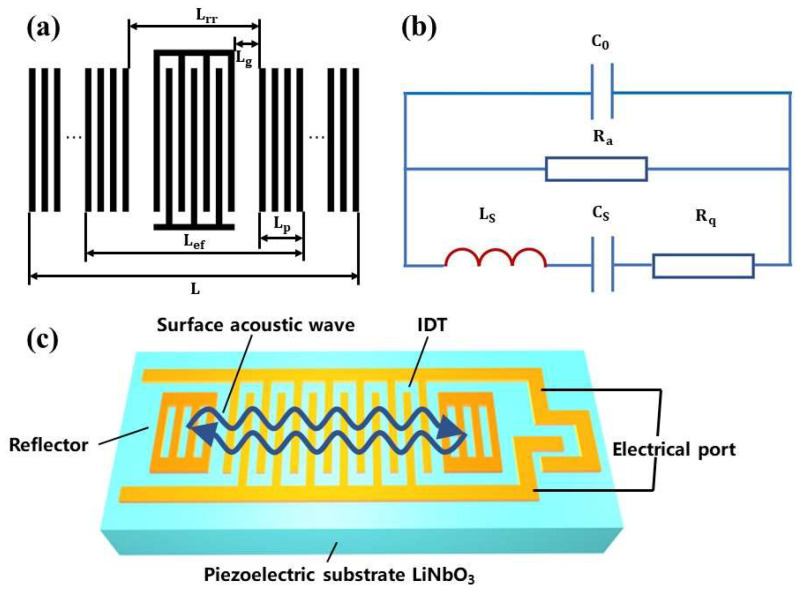
(**a**) Parameter diagram, (**b**) general equivalent circuit diagram, and (**c**) diagram of single port SAW resonator.

The operating principle of SAW devices is based on the direct piezoelectric effect and inverse piezoelectric effect of piezoelectric substrate materials. It could be seen as an equivalent circuit when the device is resonating, which converts crystal substrate and device parameters to RCL circuit shown in Figure 1b. Where $C_{0}$ is static capacitance, $R_{a}$ is radiation resistance, $C_{S}$ is equivalent dynamic capacitance, $L_{S}$ is equivalent dynamic inductance, and $R_{q}$ is equivalent dynamic resistance.

(1) $$C_{0}=N_{p}(\varepsilon_{r}\varepsilon_{0}+\varepsilon_{0})W$$

where $N_{p}$ is pairs of IDTs, W is acoustic aperture, $\varepsilon_{0}$ is dielectric constant under vacuum, and $\varepsilon_{r}$ is relative dielectric constant of piezoelectric substate. C_0_, R_a_, C_s_ and so on

The theoretical calculation formula of $R_{a}$ is as follows:

(2) $$R_{a\;}=\frac{1}{{(8K}^{2}f_{0}C_{p}{\mathrm{WN}}_{p}^{2})}$$

where $K^{2}$ is electromechanical coupling coefficient of piezoelectric materials, and $f_{0}$ is the resonant frequency of SAW devices.

(3) $$f_{0}=\frac{c}{\lambda }=\frac{c}{p_{i}}$$

(4) λ=2(a+b)

where $f_{0}$ is the resonant of the SAW device, c is the velocity of sound in the piezoelectric materials, λ is the wavelength of the SAW, $p_{i}$ is the periodicity of IDTs, a and b represent line width and gap of the IDT, respectively.

The theoretical calculation formula of $R_{q}$ is as follows:

(5) $$R_{q\;}{=R}_{a}[\frac{(1-\left|\Gamma |\right)}{\left(2|\Gamma |\right)}]$$

where Γ is reflection coefficient of reflector, $Z_{m}$ is acoustic impedance of the substrate coated surface, $Z_{0}$ is acoustic impedance of the substrate free surface, ΔZ is acoustic impedance discontinuity value of reflector, and $N_{g}$ is reflecting grid logarithm.

(6) $$\left|\Gamma \right|=\left|\frac{{\left(\frac{Z_{m}}{Z_{0}}\right)}^{{2N}_{g}}-1}{{\left(\frac{Z_{m}}{Z_{0}}\right)}^{{2N}_{g}}+1}\right|$$

(7) $$\frac{Z_{m}}{Z_{0}}=1+\Delta Z$$

The theoretical calculation formula of $L_{S}$ is as follows:

(8) $$L_{S}{=R}_{a}\cdot [\frac{L_{\mathrm{ef}}}{\left({4f}_{0}\left|\Gamma \right|\lambda_{0}\right)}]$$

(9) $$L_{\mathrm{ef}}{=L}_{\mathrm{rr}}{+2L}_{p}$$

(10) $$L_{\mathrm{rr}}{=N}_{p}\left(a+b\right){+2L}_{g}{=N}_{p}(\frac{\lambda_{0}}{2}{)+2L}_{g}$$

(11) $$L_{p}=\frac{\lambda_{0}}{4|\Delta Z|}$$

where $L_{\mathrm{ef}}$ is effective cavity length, $L_{\mathrm{rr}}$ is the distance between the two reflectors, $L_{p}$ is penetration depth of SAW energy, and $L_{g}$ is the gap between interdigital transducer and reflector.

The theoretical calculation formula of $C_{S}$ is as follows:

(12) $$C_{S}=\frac{1}{{[(2\pi f}_{0}{)}^{2}L_{S}]}$$

According to the above-mentioned equivalent circuit model and parameters of the theoretical formula, structure parameters and material characteristic parameters are closely related.

For several important parameters, the design parameters are as presented in Table 1.

There are two main types of reflectors: short-circuit reflector and open-circuit reflector [11]; ΔZ is different for LiNbO_3_, as shown in the Equation (13):

(13) $$L_{g}\{\begin{array}{c} \left(n-\frac{1}{2}\right)\frac{\lambda }{2}\left(\Delta Z\;<\;0\right)\\ \;n\frac{\lambda }{2}\left(\Delta Z\;>\;0\right) \end{array}$$

As for short-circuit reflector ΔZ < 0, and open-circuit reflector ΔZ > 0. Where n is an integer, 3, 6, 15.

Electrode material is also a vital factor for propagation of SAW. The selection of electrode material requires: substrate materials, device cycle, etc. Aluminum, copper, platinum, and gold are typical metal materials for preparation of SAW devices. Gold not only has good ductility and high temperature tolerance, but gold deposition is compatible with other micro-electromechanical systems (MEMS) [12] technology. Therefore, gold is chosen as the electrode material, which is 120 nm thick.

**Figure 2:**
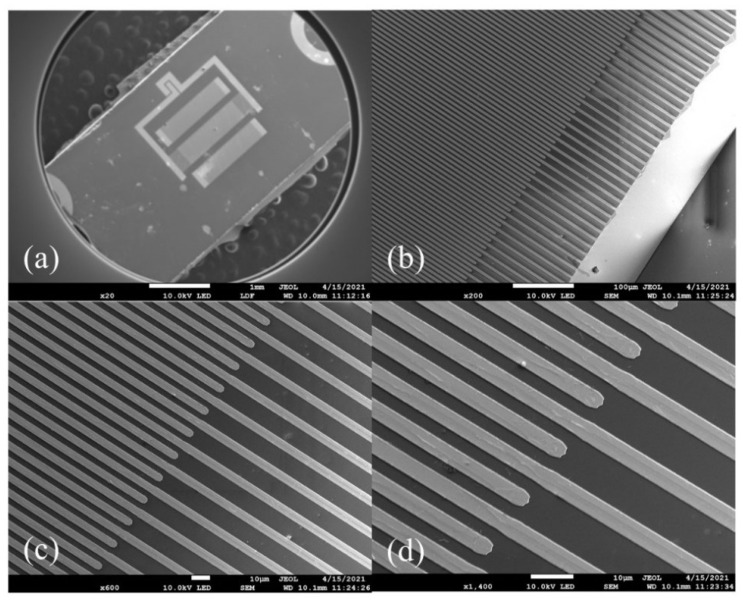
(**a**) SEM image of SAW resonator, (**b**) 200 times, (**c**) 600 times, and (**d**) 1400 times magnification of IDTs structure with a width of 4 μm.

The SAW resonators have been successfully fabricated by ion beam etching (IBE). Pretreatment is carried out primarily for cleaning the substrate, generally adopting the following processes: organic cleaning, acid picking, alkali washing. Organic matter on the surface of the LiNbO_3_ is removed by organic cleaning. The purpose of acid picking is to clean the metal particle, and the use of the alkali washing is to wipe off the acid solution residue. Then magnetron sputtering is utilized for coating the metal film. Traditional photolithography is performed on coated wafers. Finally, an IBE procession is used for removing the rest of metal. Figure 2a shows the scanning electron microscopy (SEM) image of the complete SAW resonator and Figure 2b–d exhibit the SEM images of IDTs structure with different resolutions.

### 2.2. Characterization and Testing Instrumentation

**Figure 3:**
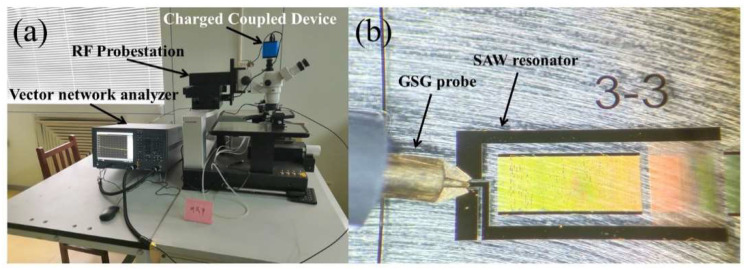
(**a**) Photo of the testing platform, (**a**) RF Probe station connected to Agilent E5071C vector network analyzer, (**b**) GSG probe with SAW resonator.

Figure 3a shows a SAW resonator test platform containing VNA and RF probe station. Microwave network parameters are tested by VNA which is Agilent E5071C made in Malaysia. RF probe station is unified with Ground-Signal-Ground (GSG) probe that has solved many measurement problems by its multiple functions and pinpoint accuracy. Figure 3b shows the image of GSG probe test under a charge coupled device (CCD) microscope.

The S11 parameter is defined as the rate of reflected power and incident power of the device which effectively captures a series of physical processes containing the acoustoelectric conversion and spreads the reflection of SAW [13,14]. The signal output and input of single-ended resonator are accomplished by the same port owing to its performance, which could be characteristic of S11 parameter. In the following section SAW resonator test results are represented by its S11 parameter.

## 3. Results and Discussions

**Figure 4:**
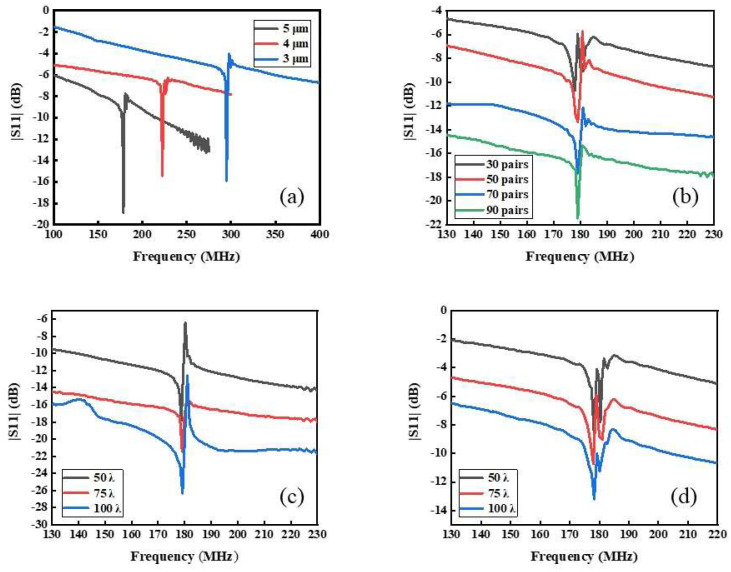
(**a**) Centre frequency of IDT with different λ, (**b**) S11 parameters curves of IDT with different pairs N_p_. S11 parameters curves of IDT with different acoustic aperture W, (**c**) each of these devices has 50 pairs of IDTs, (**d**) each of these devices has 30 pairs of IDTs. 5 µm.

According to the S11 characteristic curves in Figure 4a, the center frequencies of the SAW resonators with wavelength 12, 16, and 20 μm on 128° YX LiNbO_3_ substrate are 178.65, 222.5, and 295 MHz, respectively. The wave velocity of the 128° YX LiNbO_3_ is about 3540~3573 m/s based on the Equation (3). The periodicity of IDTs $p_{i}$ is a key factor for the resonance frequency of the SAW devices, that directly determines the resonant frequency of the SAW device [15]. To achieve a high-performance SAW device, two parameters, v and $p_{i}$ should be well considered.

The pairs of IDTs are inversely proportional to bandwidth, which means $N_{p}\propto 1/f_{\mathrm{BW}}$, and a smaller bandwidth $f_{\mathrm{BW}}$ is beneficial to improving the Q value of the device. As shown in Figure 4b, the S11 parameters of the SAW resonators with IDT are 30, 50, 70, and 90 pairs, respectively. The sidelobe of the S11 curves are suppressed and the harmonic peaks are steeply increased with the pairs of IDTs changing from 30 to 90 pairs so that the S11 gradually increases in the range of 10.725 dB. The reasonableness of $N_{p}$ should be measured comprehensively by size and difficulty of process preparation.

According to the Equation (1), an increase in W causes an increase in C_0_, which means the coupling is stronger. It is beneficial to reduce the insertion loss and improve the Q value. Figure 4c shows that the S11 parameters curves of SAW resonators with W are 50 λ, 75 λ, and 100 λ, respectively, which have the same $N_{p}$ i.e., 90. The result has an apparent tendency to increase with an increase in W, which varied from −17.38 dB to −26.32 dB. Figure 4d shows SAW resonators with $N_{p}$ of 30 and with W of 50 λ, 75 λ, and 100 λ, respectively; it can be seen that the sidelobe of the devices are suppressed with an increase of W. In brief, whether to add $N_{p}$ or increase W will greatly enhance the performance of the SAW resonators. An increase in W is accompanied by the increase in device volume. On the contrary, the diffraction effect of surface acoustic wave will be aggravated [16].

Ignoring the second-order effect, the no-load Q value of the resonator can be approximated as in Equation (14).

(14) $$Q=\frac{{\pi L}_{\mathrm{ef}}}{(1-|\Gamma |)\lambda }$$

**Figure 5:**
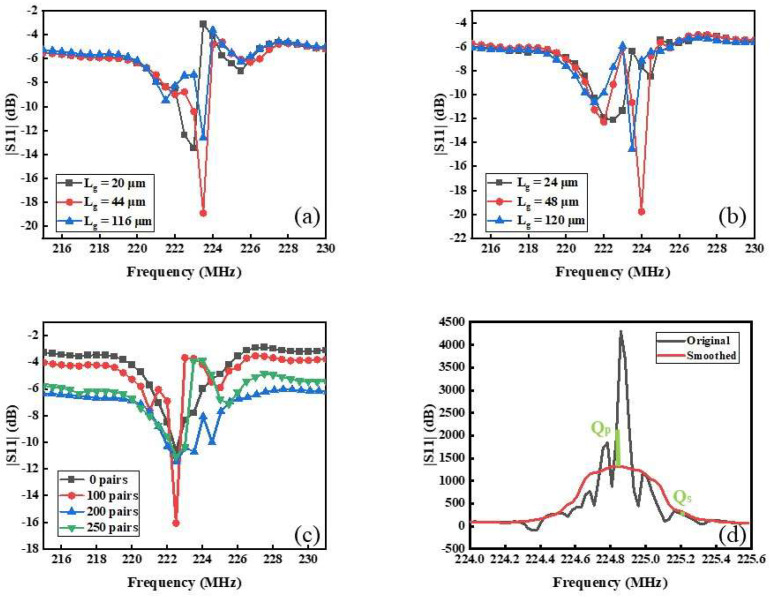
S11 parameter curves of the SAW devices with three kinds of $L_{g}$, (**a**) short-circuit reflector and (**b**) open-circuit reflector. (**c**) S11 parameter curves of the SAW devices with four kinds of reflecting grid logarithm. (**d**) The group delay algorithm calculates Q value.

It can be seen from Equation (14) that the $L_{\mathrm{ef}}$ appropriate increase is conducive to improving the Q value. Figure 5a shows $L_{g}$ = 22, 44, and 116 μm, when $L_{g}$ = 44 μm, the S11 parameter is the sharpest among them, which is −18.9 dB and its center frequency is 223.5 MHz, besides they have short-circuited reflectors. In addition, open-circuit reflectors are shown in Figure 5b. The S11 parameter of the resonator when $L_{g}$ = 48 μm is −19.78 dB which greatly exceeds $L_{g}$ = 24 and 120 μm and center frequency is 224 MHz. According to the above analysis, appropriately increasing $L_{g}$ could improve Q value. The type of reflector has little influence on the performance of the SAW resonator is yet to be researched. Appropriate $L_{g}$ keeps incident and reflected waves overlay on each other to form standing waves, that is Bragg reflection [17]. The center of the interdigital is set on the standing wave peak to enhance the electromechanical coupling efficiency [18].

Reflection coefficient has a great influence on the Q value [19], and it is positively correlated with $N_{g}$. It should be satisfied in actual design that $N_{g}\left|\Delta Z/Z\right|=3–4$. As shown in Figure 5c, the performance of the device is better when $N_{g}$ is 100, that is S11 is −16.054 dB. When $N_{g}$ is 50, 200, 250, the performance of the device is not an obvious improvement.

Group delay algorithm is adopted to calculate Q value [20,21], according to the Equation (15):

(15) $$Q\left(f\right)=\frac{2\pi f\cdot \tau (f)\cdot \left|\Gamma (f)\right|}{(1-{\left|\Gamma (f)\right|}^{2})}$$

where τ(f) is ground delay coefficient and |Γ(f)| is the amplitude of S11 [20]. The measured types for all structure SAW resonators are quite adequate for several practical designs when the pairs of IDTs are 90, the acoustic aperture is 100λ, the $L_{g}$ is 44 μm, and the $N_{g}$ is 100. The resonator shows great Q value as high as 1364.5 shown in Figure 5d, which is beneficial to manufacture highly sensitive and greater sensing sensor.

SAW resonators’ performances in previous work are summarized in Table 2, showing resonance frequency and Q value. The resonator presents the advantage of having a high Q value 1364.5, which is essential for fabricating a SAW load sensor with higher sensitivity and a larger sensing range.

## 4. Conclusions

In conclusion, a high-Q 1364.5 SAW resonator based on 128° YX LiNbO_3_ is obtained through a variety of SAW resonators with selection and optimization of structural parameters. The period of the IDT and parameter of materials determines the resonant frequency of SAW resonators. SAW resonators working on a central frequency of 178.65, 222.5, and 295 MHz were fabricated. The SAW resonators were fabricated with 30, 50, 70, and 90 pairs of IDTs, exhibiting interesting behavior to Q value. The maximum S11 shift was 10.725 dB. SAW resonators with acoustic aperture of 50 λ, 75 λ, and 100 λ were prepared and tested. Diffraction can be effectively suppressed by increasing the aperture. It is suggested that the reasonable gap between IDT and reflector was conductive to the generation of standing waves. Meanwhile, the reflecting grid logarithm increase would increase the reflection coefficient. However, the style of the reflector was not a key factor for Q value. IBE is utilized to prepare the SAW resonators and the VNA with RF probe station was used for testing them. It is suggested from the experimental results that combining typical equivalent circuit model and MEMS processing technology achieved optimal performance of SAW resonator that is of great significant for highly sensitive SAW sensors.

## Figures and Tables

**Table 1:** Main design parameters of surface acoustic wave resonator [10].

Design Parameter	Value
a = b	3 μm, 4 μm, 5 μm
N_p_	30, 50, 70, 90
W	50 λ, 75 λ, 100 λ
L_g_ (short-circuit reflector)	20, 44, 116
L_g_ (open-circuit reflector)	24, 48, 120
N_g_	50, 100, 200, 250

**Table 2:** Comparison works on performance of SAW resonators.

Piezoelectric Substrate	f (MHz)	Q Value
AlN/Al_2_O_3_ [22]	688.75	1082
Sc_0.23_Al_0.77_N/Al_2_O_3_ [23]	1910	659
LiNbO_3_ [24]	150	1150
Quartz [25]	433.05–434.79	8000
ZnO/6H-SiC [26]	688	1080
128° YX LiNbO_3_ (this work)	224.85	1364.5

## Data Availability

The data presented in this study are available in this article.
